# Editorial: Hexose Uptake and Metabolism in Immune Homeostasis and Inflammation

**DOI:** 10.3389/fimmu.2021.832293

**Published:** 2022-01-10

**Authors:** Dunfang Zhang, Chaohong Liu, Hiroko Nakatsukasa, WanJun Chen

**Affiliations:** ^1^ Department of Biotherapy, State Key Laboratory of Biotherapy and Cancer Center, West China Hospital, Sichuan University, Chengdu, China; ^2^ Department of Pathogen Biology, School of Basic Medicine, Tongji Medical College, Huazhong University of Science and Technology, Wuhan, China; ^3^ Department of Microbiology and Immunology, Keio University School of Medicine, Tokyo, Japan; ^4^ Mucosal Immunology Section, National Institute of Dental and Craniofacial Research (NIDCR), National Institute of Health, Bethesda, MD, United States

**Keywords:** glucose, mannose, fructose, autoimmunity, glycometabolism

Hexoses, especially glucose, are the major energy source of most living organisms. Many studies have shown that hexose uptake and metabolism is important in mediating immune responses. For example, the activation, differentiation, proliferation, and function of both innate and adaptive immune cells are dramatically depends on glucose uptake and glucose metabolism (1, 2). Moreover, hexose uptake affects more than only glycometabolism. One recent study showed that high fructose intake could reprogram glutamine-dependent oxidative metabolism to enhance Lipopolysaccharide (LPS)-induced inflammation in mononuclear phagocytes (3). On the other hand, hexoses may also mediate immune responses through metabolism-independent mechanisms. For example, high glucose intake has been shown to exacerbate inflammatory bowel disease (IBD) and experimental autoimmune encephalomyelitis (EAE) through the induction of T helper 17 (Th17) cells and the modification of gut microbiome (4, 5). Interestingly, not all hexoses are pro-inflammatory. Mannose, C-2 epimer of glucose, has been shown to suppress inflammation through the activation of transforming growth factor β (TGF-β) and the induction of Foxp3^+^ regulatory T cells (Treg cells) (6). These new findings demonstrate that our understanding regarding sugar uptake and metabolism in immune homeostasis and inflammation is still very limited. Thus, the goal of this research topic is to address the most recent updates on the role of sugar intake and metabolism in immune regulation. To this end, we hosted seven original research articles and mini reviews.

During hexose metabolism, hexose could be used for glycolysis, pentose phosphate pathway (also called the phosphogluconate pathway), and glycosylation. Glycosylation is a key post-translational modification in both membrane and secreted proteins that maintains their structure and function (7). Yang et al. performed precision N-glycoproteomic profiling to identify the glycoprotein variation of peritoneal macrophages in the presence of LPS, HSV, and VSV, and they identified 8326 intact glycopeptides among 587 glycoproteins. Their data suggested that N-glycosylation might be critical for the Toll-like receptor pathway. They have also demonstrated that the N-glycan on Toll-like receptor 2 (TLR2) is crucial in its transportation to the cell membrane.

Zhang Q et al. reviewed and discussed the recent advances in glucose metabolism and tumor-associated macrophages (TAMs). They focused on the modifications of TAMs that consistently occurred during the glucose metabolism process, and discussed the potential implications for TAM-based therapies in different kind of cancer. The authors also discussed glucose metabolism in the M1 and M2 types of macrophages and highlighted that metabolic change of macrophages is a critical modulator in the immune responses, not just the result of the inflammatory response.

Huang et al. reported that BMP4 could suppress CD4^+^ T cell glycolysis during T cell activation. They found that BMP4 downregulated naïve CD4^+^ T cell activation and suppressed the differentiation of IFN-γ producing T cells (Th1 cells) through Treg cell independent mechanisms. Since it has been well proven that glycolysis is indispensable for Th1 cell differentiation and IFN-γ production in CD4^+^ T cells (8), the authors proposed that BMP4 suppressed T cell activation and IFN-γ production by reducing glycolysis of T cells *via* regulating hypoxia-inducible factor (HIF)-1α.

Long-term high level hexose intake has been shown to cause insulin resistance and type II diabetes mellitus (9). Muñoz et al. reported that physical exercise-induced up-regulation of RhoA-ROCK2 signaling in skeletal muscle was associated with the increase of systemic insulin sensitivity in obese mice. They showed that RhoA-ROCK2 signaling could be a potential target to improve insulin sensitivity and maintain immune homeostasis in obese individuals and diabetic patients. Diabetes is the leading cause of chronic kidney diseases (CKD). Nakagawa et al. discussed the role of fructose in CKD. They proposed that both excessive intake of dietary fructose and endogenous fructose production driven by renal ischemia or increased glucose trafficking could mediate a metabolic switch toward glycolysis, and the increased glycolysis initiated chronic low-grade inflammation in CKD.

As high level hexose intake could exacerbate colitis (4). Zhang X et al. investigated the effect of glucose and fructose on barrier functions and inflammatory status in gastrointestinal (GI) tract and on the cecal microbiota composition. They found that glucose-fed mice, not fructose-fed mice, developed a marked increase in total adiposity, glucose intolerance, paracellular permeability in the jejunum and cecum, although both glucose and fructose intake were associated with an increase in *Il13*, *Ifng*, and *Tnfa* mRNA levels. They highlighted the deleterious effects of glucose on gut barrier function and suggested that glucose-induced increased abundance of *Desulfovibrionaceae* and *Lachnospiraceae* might play a key role in the onset of GI inflammation.

Unlike glucose and fructose, mannose has been shown to have immune regulatory functions (6). Zhang W et al. summarized studies showing the therapeutic effects of mannose treatment in both inflammatory disease suppression and treatment. They highlighted that mannose could suppress inflammation through the induction of Treg cells and the suppression effector T (Teff) cells, and mannose could also suppress macrophage-mediated inflammation by reducing IL-1β production. Although the functions of mannose in mediating anti-inflammatory gut microbiome need to be investigated further, the authors highlighted that mannose treatment is a promising novel strategy to suppress inflammatory diseases.

In summary, this Research Topic highlights the current advances regarding the hexose uptake and metabolism in immune homeostasis and inflammation. More and more evidence shows that targeting hexose uptake and metabolism in immune cells could be a promising strategy to maintain immune homeostasis and to treat inflammation and cancer. This is well reflected by the seven submissions to the Research Topic.

## Author Contributions

DZ wrote the manuscript. All authors reviewed and edited the manuscript, and approved it for publication.

## Funding

DZ is supported by the National Natural Science Foundation of China (NO. 82171829, 81600876), the Key Project of the Science and Technology Department of Sichuan Province (NO. 22GJHZ0141), the 1·3·5 Project for Disciplines of Excellence, West China Hospital, Sichuan University (NO. ZYYC21012), and the Fundamental Research Funds for the Central Universities (20822041E4084). WC is supported by the Intramural Research Program of the U.S. National Institutes of Health (NIH), NIDCR.

## Conflict of Interest

The authors declare that the research was conducted in the absence of any commercial or financial relationships that could be construed as a potential conflict of interest.

## Publisher’s Note

All claims expressed in this article are solely those of the authors and do not necessarily represent those of their affiliated organizations, or those of the publisher, the editors and the reviewers. Any product that may be evaluated in this article, or claim that may be made by its manufacturer, is not guaranteed or endorsed by the publisher.
